# Extracts from an Essay on Diphtheria and Diphtheritic Affections

**Published:** 1860-11

**Authors:** A. Jacobi


### EXTRACTS FROM AN ESSAY ON DIPTHERIA AND DIPHTHERITIC AFFECTIONS.

BY A. JACOBI, M. D.

What is diphtheria?

Diphtheria, or diphtherite, is, in its broadest sense, and from a merely anatomical point of view, a morbid condition recognizable by the exudation of fibrinous pseudo-membranes. They generally coincide with pernicious forms of inflammation, and are, in the majority of cases, exuded after a catarrh has preceded. The pseudo-membraneous exudation consists of fibrine, and is generally accompanied by serous secretion in its neighborhood, and hyperæmia and swelling of the surrounding parts. The microscope shows it to be an amorphous and homogenous mass, usually interspersed with imbedded epithelial cells, and by multiform, sometimes spherical, sometimes angular, corpuscles of mucous. The exudation is tough and elastic, or soft and pulpy, or friable, sometimes very apt to macerate, and very much like a serous liquid. It is usually membranous, expanded over a plain surface, sometimes extending into the subjacent tissue, and in some few cases forming hard lumps, of irregular shape, of a fibrinous mass imbedded in the organic tissue. Its composition is proved to be fibrinous by chemical tests. In this respect it is like the plastic exudation into the bronchi and lung cells observed in pneumonia, where fibrinous coagulations are sometimes expectorated together with bronchial mucous.

The pseudo-membranes differ in size, thickness and color, as well as in consistency. Their shape is very multiform; some are round, some angular, some regular, some irregular; their thickness varies from a transparent thinness to a quarter of an inch and more; their color is white, glassy, greenish, grey, yellowish, reddish, brown, according to their thickness, exposure to the air, and admixture of blood; sometimes unaltered blood adhering to its lower surface. They are either merely adherent to a mucous membrane, without any alteration of its tissue; such is usually the case on the mucous membrane of the bronchi and trachea, and mostly on the vellum palati. Or they are imbedded in its substance, as mostly on the tonsils and the posterior wall of the pharynx, and frequently in the larynx. It is also a very remarkable fact, that the same continuous membrane will arternately be readily removed from the surface of the mucous membrane, and again at a very distance tear the substance of the mucous membrane at every attempt at separating it. The surrounding parts are hyperæmic, and swelled by œdema during life; at post mortem examinations the œdematus swelling is sometimes found, but the hyperæmia is no longer met with after the refrigerating and contracting influence of the atmosphere has had time to operate. Pseudo-membranes are found in the pharynx, on tonsils, uvula, and vellum, on the gum, lips, and tongue, on the mucous membrane of the mouth and nose, in the larynx, trachea, and and bronchi, in the superior part of the œsophagus, in the lower part of the intestinal canal, round the anus, in the vagina, the external ear, the naso-lachrymal duct, on the conjunctiva, and on the cutis wherever and by whatever cause it has been deprived of its surface; sore nipples, etc. In all these places the chemical as well as the microscopical constitution of the the membranous exudation is entirely the same.

It is a remarkable fact that the majority of inflammatory processes attended with membranous exudation take place in the pharynx; cases of both sporadic and epidemic pharyngeal diphtherite will sometimes be accompanied with diphtheritic processes of other parts; but diphtherite of the external ear, or vagina, will but seldom be heard of without being accompanied with pharyngeal diphtherite. The few cases of this kind observed by us we count among exceptional curiosities. Next to pharyngeal diphtherite, in frequency, comes the mucous membrane of the respiratory organs, especially of the larynx and trachea, and nostrils. Sometimes the exudative process will either extend from one part to the adjoining one, or will merely wander; sunden transmission to distant regions, or contemporaneous affection of distant parts, being usually not observed, except in epidemics of diptheritic affections. To point we shall return at some future occasion.

We have stated that the majority of diphtherites are met with in the throat. Thus it occurs that the term of diphtherite is usually applied and attributed to the peculiar process of exudation in the fauces; so much so, that we generally have forgotten that dysentery, croup, etc., consist in, and show, the same anatomical lesions as diphtheritic sore throat; the difference of accidental symptoms and our tendency to classification either preventing our perceiving or acknowleding the equality in what simulates differences. Certainly, the same physiological or pathological process, in different organs, ought to be understood to offer a series of different symptoms.

Our past epidemic has again proved that diphtherite is, in almost all cases, confined to, or originates in, the throat. Thus, diphtherite and diphtheritic sore throat are almost, as it were, identical terms. At all events, the presence of membranes in the throat may be taken as the ordinary occurence; and in the following symptomatology we speak particular regard to this usual form of diphtheria.

A child shows symtoms of moderate fever; pulse accelerated, perhaps a little small; face somewhat flushed, submaxillary region a little swelled to both sight and touch; more or less headache is complained of, swallowing has been interfered with some days; tongue has a soft, creamy-whitish, or whitish-grey or yellow fur, diminishing in thickness towards the lateral, and particularly the anterior region, where the papillæ appear a little enlarged and higher colored. One or both tonsils, or the posterior wall of the pharynx, the velum, uvula, or some of the enumerated parts at the same time, are covered with a membrane as described above. The surrounding mucous membrane looks red, livid, is hyperæmic, and shows œdematous swelling. The submaxillary and cervical glands are a little swollen. The breath of such patients has generally a bad smell, although the membrane does nor extend very far; at all events, some smell will be perceived as soon as the membrane begins to macerate or fall off; especially is this the case when the throat is not kept clean. Such are the average of mild cases, and the great majority of all those which come under observation in the onset of an epidemic. They will get well in five, six or eight days; some spontaneously, some after appropriate treatment. The membrane will be removed as a whole, and leave the mucous membrane underneath smooth, livid; or it will macerate, fall off in pieces, or be removed as a half-serous liquid, and leave the mucous membrane, especially of the tonsils, with a ragged appearance and superficial ulcerations. At the same time, the submaxillary and cervical glands will return to their normal size.

A number of cases occurred during the epidemic, in which all the symptoms were present, with the exception of the formation of the membrane; there were some amongst them in which the symptoms of fever and adenitis, and even a general adynamic condition, were well pronounced. In such cases never thought of putting the case down as diphtheria, but recorded it as pharyngitis, amygdalitis, or stomatitis: a number of cases which we, according to the severity of symptoms, supposed to be of a diphthetic nature, we put down as diphtheritc pharyngitis, or diphtheritic fever; the former name being selected for such cases in which the symptoms of pharyngitis prevailed, the latter for such as showed fever as their foremost symptom. We were seldom mistaken, as in a day or two membranes would generally present themselves.

There is a form of the diphtheritic process, in which very little or no fever is perceived, and little or no glandular swelling will take place. The congestion and swelling of the pharynx is not very remarkable, and the first remarkable appearance is noticed on the follicles of the mucous membrane of the pharynx: they are visible as whitish grey spots of a twentieth or twelfth of an inch in diameter. Not long after, however, membranes are formed, and the whole process will run its course in sometimes three or four days, without any great inconvenience to the patient. But there are cases in which the symptoms will increase in severity, fever will set in, and submaxillary and cervical adenitis take place. Such cases have been separated by some authors as "common membranous angina," as "herpes of the throat," as "herpetic angina." We do not see anything else in these cases but light diphtherite, mostly without well pronounced general symptoms. We have not found any more reason to distinguish this form, of which, however, we have not seen more than a dozen of cases from diphtheria, than we should think of excluding a case of scarlatina, without fever, and with less than usual eruption, from the record of cases of scarlatina. Moreover, we have pointed to the fact that such apparently simple cases will sometimes be followed by fever and adenitis; and when we further add that some of these light cases of "herpetic angina" have been followed by diphtheritic paralysis, we ought to lay aside our fondness for classification and subdivision; and take nature as a whole, the different pathological conditions of the diphtheritic process are variable in their appearance, but alike in their innermost nature.

The local process of exudation is not confined to a certain limit. Membranes will be found on the posterior wall of the velum, and in the posterior nares, blocking up the nostrils and extending as far down as the lips. In such cases small children, who are mostly used to breathe through the nose, suffer much from dyspnœa. These membranes will be removed in the course of time, and with the same consequences as those of the pharynx itself. To one peculiarity, however, we wish to direct the attention of our readers. We have once seen, in consultation, a little girl of five years, in the upper part of the city, with extensive membranes of the pharynx, velum, etc., but were struck with the fact that long cylindrical tubes of false membrane were brought up. These cylinders could come from the trachea only; how was it that the child was not suffering all the time from the utmost dyspnœa, but felt proportionately not bad and gave, in its general appearance, a good prognosis? We learned from our friend Dr. S., who was then suffering from extensive diphtheria himself, that he, too, had brought up cylindrical tubes of membranes like those thrown off in children suffering from laryngeal and tracheal diphtherite, but that in his case they came from the posterior nares. We desire to state this fact, supposing that some professional man might in some case have the same difficulty in explaining the occurrence of cylindrical tubes of membrane that do not come from the respiratory organs.

In other cases the exudation of pseudo-membrane will extend downwards instead of upwards, and will then constitute what is generally called croup. The diagnosis of this descending croup is difficult in one respect only, viz : The œdematous swelling surrounding the membranous exudation will sometimes produce croupous cough and dyspnœa, etc., before the membrane has reached the larynx, by œdema of the epiglottis or glottis, or general laryngeal catarrh. As a general thing, there is not much practical difference, for in the majority of such cases the process is of such rapidity, that we need not wait long for real membranous exudation. At all events, the symptoms of this descending croup of the diphtheritic process are the same as are known in genuine or sporadic croup; and therefore, after we have learned besides, that the anatomical elements and constitution in diphtheritic membranes of the pharynx and larynx are the same, we have a right to say that there is no real anatomical difference between "diphtheritic" and "genuine" croup. We need not return to the consideration of the former assumption of a difference between croupous and diphtheritic membranes. It does not exist. Croupous membranes were those which were stretched over the mucous membrane, while the name of diphtheritic membranes was given to such as would be imbedded in the membrane and leave ulcerations, or loss of substance, when removed. There is no such difference.

There have certainly been cases of croup, genuine croup, differing widely from each other, in the experience of every one of our readers. The differences are either of an individual or of a general nature. The former do not interest us here, the latter do. Genuine croup is apt to cause death either by suffocation, or by poisoning with carbonic acid; and diphtheritic croup may show a third and fourth cause of death, viz: exhaustion, and diphtheritic poisoning. And it is here that we wish to add some remarks on the true nature of diphtheria, as shown by observation and rational conclusions.

Diphtheria is a general disease; it has local deposits, it is true, but in the same manner that scarlatina will localize itself on the skin, mucous membrane of the Bellinian canals, etc., measles on the skin, mucous membrane of the respiratory organs, etc., or typhoid fever on the mucous membrane of the intestinal tract, etc. Thus diphtheria will, being eminently a constitutional disease, localize itself on the mucous membranes and denuded skin in general, and on the mucous membrane of the pharynx and larynx in particular. Therefore, the danger of the disease depends by no means on the extension or thickness of the exudation, sometimes a very small amount of exudation taking place in extremely dangerous cases. Some cases have set in with an exceedingly high fever; with vomiting, which so generally is a symptom of sudden and general affection of the system; with convulsions, like those observed at the outbreak of acute exanthems, and with rapid collapse not at all explained by any of the visible symptoms.

Although not all the fatal cases ran their course with this entire absence of positive symptoms, terminating in death, as it were, without disease, a great number of cases have come under my observation, in which the local exudations were by no means in proportion to the general symptoms, and to the character of the attack. Excessive fever, with a pulse of 140, 150, 160, like that in scarlet fever, but generally of a more adynamic character, and large swellings of the submaxillary and cervical glands have, in a large number of cases, been observed to precede the exudation of fibrinous membrane for three, four, even five days. Then, at last, membranes would make their appearance, and either cover, in some instances, the pharyngeal mucous membrane and the adjoining parts to a large extent, or, in most cases, remain for a time of moderate size. Such cases are never without danger; the adynamic character of the disease, combined with the intrinsic danger of a fever like that described, being fully able to produce death from exhaustion in a very short time indeed. Such have been those cases also in which a septic character would develop itself. Instead of being removed as a whole, or in pieces, the membranes would decay, macerate, or flow off, with the serous secretion of the adjoining mucous membranes, as a thin, sanious matter, of watery color, and excessively foul smell, and would exulcerate every healthy part it came in contact with. Ulcerations, on the original seat of the membranes, or such as were successively formed on neighboring localities by contact with the sanious, foul secretion, would again prove the seat of new secretion and stench, and sometimes require many weeks, even months, in such individuals who suffered most from anæmia and exhaustion, to perfectly heal. We will here refer particularly to those cases of which a large number have been observed. In a family where diphtheria was just reaping its harvest, or in the neighborhood, children would fall sick with all the initial symptoms of diphtheria, excessive fever, exhaustion, pharyngitis, and adenitis, but no membranes would appear. The whole would take a course like that of diphtheria, and the patients require a long time to recover. Such cases we have always put down as diphtheritic fever. We feel sure, however, that they were entitled to be named diphtheria like those in which membranes were formed. Similar cases will occur in other epidemics, where part of the symptoms only will be fully developed, without, however, losing their general character; there is a great difference, too, among cases of scarlatina in every epidemic, according to individual nature, accompanying circumstances, and the severity of original infection or contagion. Further, there is a great difference, depending on whether a case of zymotic disease will occur sporadically, or during the height of an epidemic; sporadic cases, and such as occur at the commencement of an epidemic, generally proving to be milder forms. Thus diphtheria, in sporadic cases, or in the beginning of the epidemic, may look like a merely local disease, being, in its nature, a general affection of the system. Diphtheria, with its local exudation into the larynx has, for many decenniums, generally been observed sporadic, and therefore has been taken to be a merely local affection. It has been otherwise in our epidemic. At all events we believe we have shown that pharyngeal as well as laryngeal diphtheria may have the appearance of either a local alteration or a constitutional disease; its true nature being general and constitutional, like scarlet fever, or any of the diseases comprehended under the head of zymotic affections. At this point, finally, we allude to the real meaning of the terms of "diphtherite" and "diphtheria." According to its origin the former means a local, the latter a general affection. If a uniform name was to be given to all the cases occurring, both sporadically and epidemically, it could be that of diphtheria only; but such cases as exhibit more of a local character, might as well be called pharyngeal, laryngeal, cutaneous, etc., diphtherite.

We have a few more words to say on the swelling of the submaxillary and cervical glands. It is a symptom that will be found in almost every case, and never missed in any severe one. It is so certain to be met with, and so little, in many cases, in proportion to the extension of the membranes, and, moreover, so generally keeping pace with the danger of the fever, that we have been taught to consider the amount of glandular swelling accompanying pharyngeal diphtheria, as a highly valuable prognostic symptom. It will often remain after the other symptoms have subsided, but the case must not be considered as perfectly safe, as long as the glandular swelling or induration lasts. Suppuration of the diseased glands we have met with in very few cases. We consider the glandular swelling as a means of determining the amount of constitutional infection.

The epidemic nature of the disease is an acknowledged fact; consequently we do not wish to carry owls to Athens by repeating what every reader knows. Nor is it necessary to say much on communicability of the disease by endemic influences. For every practioner who has but seen a limited number of cases, has certainly learned, that cases will seldom occur isolated in one locality. Generally, a number of inhabitants of the same room, house, or neighborhood, will be affected at the same time, or in short intervals. Thus hospital wards containing cases of diphtheria, will soon exhibit a number of other cases of the same disease. This fact has been so reported, and is so well known, that this source of infection need not be dwelt upon any longer. But the question arises, whether many cases believed to be the result of the same general endemic influences, exercising their power on every inhabitant alike, have not rather been the consequences of direct contagion. This question of the contagiousness of diphtheria has been the stumbling block of most writers, and, we must confess, of some of the most conscientious ones. For certainly the more scrupulous an author would be, the more he will feel bound to restrict the territory of contagion in all cases that give a chance of being explained by endemic influences alone. For our own part, we at once state our conviction of the contagiousness of diphtheria. We know full well that the proof is not easy. Inoculation has proved either fruitless or improbable; the cases of surgeons related to have been directly affected by diphtheritic patients, admit of other explanations; the vast majority of cases believed to be, and reported as proofs of contagiousness, are really better explained by local endemic influences.

* * * * We do not mean to say that it necessarily must be communicated to everybody coming in contact with a patient—although we have repeatedly observed such members of a family as were most engaged in waiting upon those already affected, to be the next sufferers—just as little as we assert that the like endemic cause existing in a room, or house, or neighborhood, must necessarily strike down every human being under its influence. Nor do we think that no case of diphtheria could originate in any other way except by contagion. But we feel sure, that it partakes of the nature of such epidemic diseases as typhoid fever, or scarlatina, or measles. The failure of experiments is no proof; for when has the direct experiment on the contagiousness of typhus, scarlatina, and mesles, or on their inoculability, given any satisfactory result? And nevertheless, although there are some who deny contagion by typhoid fever, all three are recognized to be propagated by direct communication from one individual to another. The diphtheritic membranes, moreover, observed to form on the sore nipples of mothers, wet nurses, whose children suffered from diphtheria, appear to be as good a proof that contagion is a cause of diphtheria, as any that could be found in any other disease.

The complications of diphtheria are very numerous. Nothing else can be expected in a disease which is as apt to last a long time as it will generally impair health and nutrition for a protracted period. We do not count coryza, croup, etc., mere localizations of the disease, amongst its complications, nor shall we have anything to say on hemorrhages occurring during its course until we enumerate its final consequences; nor do we feel sure whether we are right in considering pneumonia, with its rapid exudation, as a mere accidental complication; for it is certain that pneumonia will very often occur during the diphtheritic process, and may easily be influenced by the peculiar constitution of the blood giving rise to rapid and copious fibrinous exudation. It is well known to all of our readers that croup will oftentimes be complicated with pneumonia, and even prove fatal not by its own influence, but by the sudden and extensive propagation of the exudative pro cess to the bronchi and lungs. Whichever opinion may be right, it is certain that pneumonia is very frequently met with in company with diphtheria. We have further noticed as complications, of more or less accidental nature, several zymotic diseases, as scarlatina, measles, urticaria, furunculosis, erysipelas, intermittens, and intermittent cephalalgia, varioloid and acute rheumatism; further, general anemia, paralysis, (pre-existing,) rachitis, pulmonary tubercles, eczema of the face, dysuria, stomatitis aphthosa, and ulcerativa, gangrene of the throat and noma; gastric, intestinal and gastro-intestinal catarrh; laryngeal catarrh; dilatation and hypertrophy of the heart; hyperæmia of the brain and meningeal œdema. Many of these complications are very dangerous and frightful indeed. We have attended a young man of nineteen years, residing in Christie street, in whom the pharynx and nostrils were the seat of diphtheritic membranes; adenitis and fever being in the beginning very moderate. The membranes were hard and thick, and proved very obstinate for some weeks, until, in February, the patient was affected with varioloid. From this time the strength of the patient gave way, the fever rose, appetite disappeared, mucous membranes grew pale, skin very inactive. The usual time of desquamation passed by, but no desquamation took place; the scurfs grew hard and dry, and gave a great deal of pain on being touched; at the same time, they increased in height and diameter, so as to urge upon us the necessity of removing them by force. Beneath each of them the surface was a deep discolored ulcer, secreting a whitish yellow or greenish pale matter from a whitish gray membrane that covered the ground. Thus each varioloid pustule constituted a diphtheritic ulcer, a hundred of which covered the head and face and trunk and extremities of our unfortunate patient. It may suffice to say, that he required several months before the diphtheritic ulcers of his scalp and face got well, and that he has not recovered his former health and strength up to this moment. Now that such complications of two zymotic diseases should occur, is not at all improbable, nor are they very rare. There are many cases in which we should do well to remember that a complication of scarlatina and measles, or of measles and urticaria, form a compound that is sometimes met with in practice, and is very apt to mislead the pratitioner, and to be misconstrued by him. Nor is it an entirely extraordinary occurrence to see an acute exanthema step in before another has fully completed its course. Thus we have the case of a child, on whom we were fully able to diagnosticate, to our entire satisfaction, the presence of scarlatina, urticaria, measles, and finally varioloid, in the course of thirty two days from the first to the last attack.

Not an unfrequent complication of diphtheria is albuminuria, which may be observed in any stage of the disease. It is not at all true, that albuminuria in diphtheria and in scarlatina shows the difference pointed out by French writers, viz: that albuminaria is a complication of the first stage of diphtheria, and of the later periods of scarlatina; this assumption being equally erroneous in either of the two. About a quarter of all the cases examined for albuminuria gave a positive result as to its presence, and nearly all the post mortem examinations revealed an hyperæmic condition of the kidneys; this being the first cause of, or at least co-existent with, albuminuria. Thus it appears that albuminuria, although found in many cases of no severity, nor sometimes attended with any danger, is a complication of more than average importance.

The etiology of diphtheria shares the fate of that of all the zymotic diseases of being exceedingly obscure. It is an endemic as well as epidemic disease, and will therefore be found with every constitution, amongst all classes, in either sex, at all ages, in all climates, and at all seasons. But some differences are found that modify our assertion to some extent. The first four or five months of both years, 1859 and 1860, show by far the largest number of cases. In our cases, besides, the male sex was in a slight but decided majority throughout the whole course of the epidemic. Age, too, shows differences similar to those exhibited for scarlatina and measles. The two hundred cases enumerated above are of children under fourteen years of age; the number of diphtheritic cases beyond this age, in the same institution, for the whole time, is from thirty to thirty-five; moreover, the large majority of those two hundred cases occurred in children from two to five years of age, the average age being three years. Cases under a year are not frequent, over nine or ten years proportionately rare. We do not remember to have seen a patient suffering from diphtheria at the age of more than fifty years, but have been told of some few occurring between seventy and eighty. The majority of cases were children with impaired constitution, badly fed, and with bad digestion, anæmic or suffering, or convalescent from another disease that diminished the amount of the solid elements of the blood, and the strength and power of resistance. Among the most unpropitious accompanying or preceding diseases are scarlatina and measles, which generally give a pretty unfavorable prognosis when existing with or followed by diphtheria. Scrofulous individuals, with catarrhal affections of the mucous membranes, of long standing, are particularly subject to being affected, and thus, undoubtedly, poverty, dirt, and a want of care, have a great influence in producing this and other general diseases. Dense population must be accused in many instances, firstly, because of its very co-existence with poverty, want of fresh air, and uncleanliness; and secondly, because of the readiness of communication by direct contagion. But it is nevertheless a fact, that many cases, and many dangerous and fatal ones, occurred in healthy and robust children, wealthy families, good situation, and fresh air.

(Concluded next month.)

